# Factors Affecting Performance in Clinical Practice among Preservice Diploma Nursing Students in Northern Tanzania

**DOI:** 10.1155/2019/3453085

**Published:** 2019-03-03

**Authors:** Helena Marco Gemuhay, Albino Kalolo, Robert Mirisho, Beatrice Chipwaza, Elijah Nyangena

**Affiliations:** ^1^School of Health Sciences, Kenya Methodist University, Kenya; ^2^Faculty of Medicine, St. Francis University College of Health and Allied Sciences, Ifakara, Tanzania; ^3^Department of Nursing, University of Kabianga, Kenya

## Abstract

There is an increased call for improving the environment in which nursing students learn the clinical skills. Clinical practice in the clinical placement sites should allow students to apply their theoretical knowledge in a real environment, develop nursing skills and clinical reasoning, and observe and adapt the professional role. This study aimed at identifying the factors influencing performance in clinical practice among preservice diploma nursing students in Northern Tanzania. This study relied on a cross-sectional analysis of data collected from nursing schools in Northern Tanzania in which 208 (123 nursing students and 85 nurse tutors) participants were recruited in the study. Data was gathered using a self-administered questionnaire which collected information on sociodemographic characteristics and factors influencing clinical practice categorized in students' factors, hospital based factors, social-economic factors, and nurse tutors opinions assessed. Descriptive analyses and chi-square test were employed to understand the background information of the sample and association between variables. Majority of the nursing students (84.4%) agreed that clinical placement offers students adequate opportunity for clinical practical learning. Barriers to effective clinical learning was reported by 70.1% of the participants and the barriers include student factors such as lack of self-confidence and absenteeism, school factors such as improper supervision, and poor preparation of clinical instructors or clinical facility factors. We found a significant association between type of barrier and gender (chi-square 0.786,* p=0.020)*. More male nursing students (62.1%) significantly reported unsupportive environment as a barrier and anxiety was more common in female nursing students (48.9%)* (p=0.020). *Reporting of barriers to effective clinical learning by students from different schools of nursing was not significant (P=0.696). In addition, age of participants did not have significant association with effective clinical practice (p=*0.606). *Student's factors and placement based factors played an important role to influence clinical learning experiences. Offering preclinical orientation, distributing and clarifying clinical learning objectives to students, and frequent visits and supervision of students in clinical area may improve student learning experience in clinical placement. In addition, tailoring the interventions to gender may improve learning experiences.

## 1. Introduction

There is an increased call for improving the environment in which nursing students learn the clinical skills, especially in poor resource settings. Clinical placements where students learn clinical practice should allow students to acquire nursing skills and clinical reasoning and develop as professional nurses. In areas where simulation learning is limited or not available at all, learning takes place in the real environment [1]. Although nursing education is a combination of theoretical and practical learning experiences that enable nursing students to acquire the knowledge, skills, and attitudes for providing nursing care [2], globally clinical training of nursing students is seen as the basis of nursing practice. Nursing students need to be supported and guided so that they can become responsible, accountable, and independent professionals who are able to function within the scope of professional practice [3]. Clinical practice provides opportunity for students to apply the theoretical knowledge into actual health care provision [4, 5]. Clinical placements enable nursing students to gain essential skills and provide safe and quality nursing care through real life practice [6, 7]. In the course of clinical practice, students apply their theoretical knowledge in a real environment, develop psychomotor skills, and observe and adapt to the professional role. The clinical environment should be carefully selected, accepted by nurse tutors, and prearranged to be capable of transforming nursing students into competent nursing practitioners [8, 9]; this is dependent on factors such as curriculum design, cost effectiveness, and relationship with specific health facilities [10].

The environment where nurses learn clinical practice, especially in sub-Saharan African settings, is challenged by multitude of factors which can affect learning adversely. Some factors reported to affect students learning in clinical area act at different levels namely individual level (student factors), hospital environment, social-economic factors, and nurse tutors factors [1]. A Study conducted in Botswana revealed that nursing students failed to apply theory into practice because they lack adequate supervision in the clinical area which results to low performance in their clinical practices [9]. It has been reported that clinical instructors' attitudes affect performance of nursing students in the clinical area. Positive attitudes and supportive environment encourage students learning environment. Constructive criticism improves practice in the clinical setting while negative criticism is an obstacle for effective clinical performance. Limited chance for hands on practice in training hospitals, shortage of nurse tutors, clinical instructors, and too many students in the program can negatively affect performance of students in clinical practice [11, 12]. The students' behaviors negatively affect students' performance in clinical practice as inability to demonstrate knowledge and skills, attitude problems, unprofessional behavior, and poor communication skills with patient and clinical instructors, not asking questions, overconfidence, unmotivated to learn or to work, lack of confidence, and dishonesty [13]. Lack of prior clinical experience, unfamiliar areas, difficult patients, fear of making mistakes, and being evaluated by faculty members were expressed by the students as anxiety-producing situations in their initial clinical experience [14]. Initial clinical experience was found to be the most anxiety-producing part of their nursing education. The sources of stress during clinical practice have been observed by many researchers and includes new environment, lack of assessment before supervision, poor clinical orientation, and shortage of nurses [15]. Stress or anxiety is not only a workplace problem but different stressors can also affect the students learning [16].

In Tanzania, nursing education is offered based on the requirements set by the Tanzanian Nursing and Midwifery Council (TNMC) and regulatory authorities for academic award mainly The National Council for Technical Education (NACTE) for diploma programs and Tanzania commission for Universities (TCU) for bachelor's, master's, and doctoral programs. The training programs offer theoretical and practical learning for general nursing and midwifery. Tanzanian Nursing and Midwifery Council (TNMC) is a professional regulatory authority responsible for registration of who have completed and passed the 3 years diploma program and licensure examination and are permitted to take the licensing program to become Registered Nurses (RNs).

Although there are a number of studies which have identified factors affecting learning among nursing students, only a few have been conducted in Tanzania and majority have focused specifically on those factors affecting performance of nursing students in general [11]. Scarcity of studies that explain the reasons for observed heterogeneity in performance of nurses in clinical settings warrants a study like this to address this knowledge gap; our study aimed at assessing the factors affecting performance in clinical practice among preservice diploma nursing students. The findings of this study may generate results that could influence policy, nursing practices, and teaching institutions to recruit more nurse tutors and clinical instructors in Tanzania and beyond.

## 2. Methodology

### 2.1. Study Design

This study relied on a cross-sectional analysis of data collected from nursing schools in Northern part of Tanzania. The study included sampling of nursing schools to participate, selection of participants, collection of data, and analysis of the collected data.

### 2.2. Study Setting

Tanzania is a low-income country with a population of 44,928,923 people where approximately 75% of the people live in rural areas. Tanzania economy is dependent on agriculture, which employs almost 80% of the population. Tanzania experienced economic growth of between 5% and 7% since 2000 to 2007 per year until the global financial crisis in 2009. However by now it has been regained to 7%. The poverty rate in Tanzania stands at 33.4% and the country's capacity to collect taxes remains low and hence depends on donor funding for about 29% of total country budget [18]

**Figure 1:**
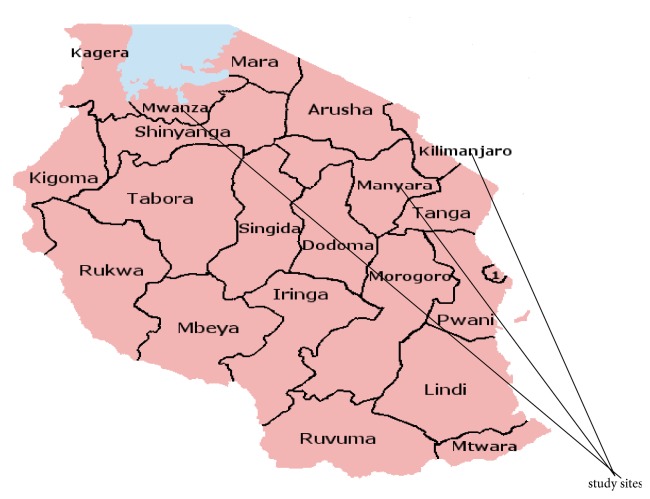
Map of Tanzania showing the location of study areas [17].

The three regions (Kilimanjaro, Mwanza, and Manyara) have a total of 6,837,727 people which forms about 14.8% of the Tanzanian population. The three regions have a total of eight schools of nursing that offer a diploma in nursing which were all included in this study (Figure 1).

The number of students per school ranged from 120 to 220 and number of tutors ranged from nine to twelve. The distribution of students in the respective regions is detailed in Table 1.

The teaching hospital for each of the eight nursing school was the nearby hospital which had a status to act as a teaching hospital. The teaching hospitals varied from one school to another where some were zonal referral hospitals, regional referral hospitals and district hospitals. The hospitals are owned by faith based organizations (FBO) but receive some support funding from government through public-private partnership agreements. Details of each hospital are provided in Table 2.

### 2.3. Sample Size

The total sample of the study participants was 208 including nursing students and nurse tutors. The students sampled were those who have been in clinical areas long enough to give their informed views on clinical practice suggestions and were in the third year of study. The study participants were from the three administrative regions among 28 regions of Tanzania. The three regions have a total of 8 diploma nursing students within 8 districts and researchers included all diploma nursing schools within those regions in the study. According to Mugenda and Mugenda 10% of the population is adequate to represent the entire population [19]. Simple random sampling was used to select nursing students within the schools, whereas all nurse tutors were included in the study given their small number. Table 1 summarizes the above information.

### 2.4. Data Collection

The researchers developed self-administered questionnaires based on literature review [5, 20]; the questionnaire collected information on sociodemographic characteristics and factors influencing clinical practice categorized in students' factors, hospital based factors, social-economic factors, and nurse tutor's factors and their opinions. Data collection was completed over a period of 3 months. To ensure validity, the questionnaires were developed based on literature review and objectives alignment to the objectives of the study. Furthermore, specific objectives were checked against all the items in the questionnaires. Pretesting of the questionnaire had happened before actual data collection. The pretest aimed at identifying any ambiguities in the questionnaires and to correct them before administering to the research participants. The questionnaire was in English language for easy understanding for all participants.

To ensure reliability of the questionnaire, pretesting was done by administering the same questionnaire to participants who are not included in the study twice at an interval of two weeks. Both two sets of answers from the same group of people were scored and Pearson moment correlation revealed a score of 0.72 for the two groups. The reasons for doing two tests were to understand the correlation of the two tests. The reliability of each of the tests was also computed. The reliability coefficient of ≥ 0.7 was considered enough to judge the reliability of the instruments.

### 2.5. Statistical Analysis

The questionnaires were coded and numbered for easy follow-up, distributed, and collected later, once they were filled by the participants. Descriptive analysis was done to summarize information of the participants. The information was presented descriptively by frequencies and percentages while being enhanced by figures (histograms) and tables.

Chi-square was done to test the association between variables. Data was checked and cleaned for accuracy and completeness and entered into the computer. Analysis was done by STATA software version 11.0 for windows.

## 3. Results

### 3.1. Sociodemographic Characteristics of the Study Participants

A total of 96 nursing students from 8 schools of nursing filled and returned the questionnaire. The mean age was 23.3 and the median age of the students was 23 years with an interquartile range 22–24 years and the female students were 63 (65.6%) while male were 33 (34.4%). All 85 nurse tutors in the sampled schools completed the questionnaire and their mean age was 44.4 with standard deviation 6.8 years. There were 61(71.8%) female while males were 24 (28.2%).

### 3.2. Students Factors Affecting Performance in Clinical Practice

Majority of the nursing students (84.4%) agreed that clinical placement offers students adequate opportunity for clinical practical learning. The most reported factor that improved clinical practice was effective supervision and assessment as reported by 32.3% of participants. Existence of barriers to effective clinical learning was reported by 70.1% of the participants. The reported barriers include student factors such as lack of self-confidence and absenteeism, school factors such as improper supervision, and poor preparation of clinical instructors or clinical facility factors as shown in Table 3. The most reported reason for poor clinical practice was poor communication between hospital staff and students (49.0%)

Majority (60%) of nursing students reported that clinical placement did not provide them adequate opportunity for effective clinical learning and they mentioned shortage of nurse tutors in clinical area as the main reasons for inadequate clinical learning (60%) followed by learning resources (26.7%) and inadequate supervision (13.3%) as illustrated in Table 4.

The most reported barriers preventing effective performance in clinical practice leaning were unsupportive environment due to shortage of health care staff in the clinical placement sites, lack of clinical instructors and nurse tutors, high patients loads for staff in the ward 34 (45.9%) and anxiety 27(36.5%) among students.

**Figure 2:**
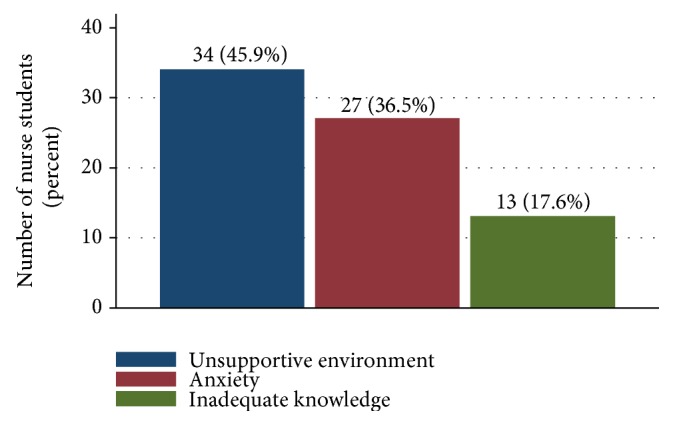
Barriers preventing effective performance in clinical practice.

Figure 2 summarizes the reported barriers preventing effective clinical practice of nurse students.

### 3.3. Responses of Nursing Students on Anxiety as a Factor Affecting Negatively Clinical Practice

Anxiety among the nursing students was related to fear of making mistakes (47.9%) and lack of competency (31.2%). The situations that mostly caused anxiety were clinical assessment during practical examinations (38.5%) and overly strict supervision (26%). Measures suggested by students that could reduce anxiety were friendly clinical teaching, adequate clinical supervision (44.8%), fair of clinical assignment, and frequent clinical case presentation (29.2%) by nursing students indicated in Table 5.

### 3.4. Association between Barriers of Effective Clinical Practice and Gender, Age, and Nursing Schools

We found a significant association between type of barrier and gender (chi-square 0.786,* p=0.020)*. More male nursing students (62.1%) significantly reported unsupportive environment as a barrier than female (35.6%). Furthermore, anxiety was more common in female nursing students (48.9%) compared to male nursing students (17.2%).

Reporting of barriers to effective clinical learning by students from different schools of nursing was not significant (P=0.696). In addition, age of participants did not have significant association with effective clinical practice (p=*0.606).*

### 3.5. Hospital Factors Affecting Performance in Clinical Practice by Students

Shortage of staff in the hospital affected clinical supervision as reported by 89.6% of the students. Furthermore 22.9% of nursing students reported lack of equipment for performing procedures within the hospital and lack of well-equipped skills laboratories as an important factors affecting clinical practice. In addition, 21.9% of nursing students reported lack of teaching/learning resources such as equipment for nursing care procedures. This means that sometimes students performed procedure by shortcut contrary to the theory learned in class as depicted in Tables 6 and 7.

### 3.6. Effect of Student's Social-Economic Background on Clinical Practices

Social and economic factors of nursing students were reported to affect clinical practice and create psychological problems. The social climate of the school was important in enabling students' clinical learning. Majority of nursing students (84.4%) agreed that parent's economic status affected clinical practice. Lack of money caused inability to afford learning materials and other personal needs and often led to unwanted pregnancy especially among some female students who are tempted to engage in sex for money as summarized in Table 8.

A positive school social environment improved clinical practice as reported by 85.4% of respondents while 20.5% reported good interpersonal relationship and cooperation among students boosted self-confidence. Other effects of a good school social environment on clinical practice are summarized in Table 9.

### 3.7. Ways of Improving Clinical Practical Learning

The approaches of improving clinical practice suggested by students include frequent use of skills laboratory (62.5%), participation in nursing conferences in the hospital (18.7%), use of simulation (12.5%), and watching nursing procedures videos to gain more skills (6.5%).

The most mentioned approaches by nurse tutors were recruitment of sufficient number of nurse tutors that matched the number of students (51.8%) and availability of modern skills laboratory for demonstration (42.4%) as summarized Table 10.

## 4. Discussion

The current study aimed at identifying the factors affecting clinical practice among nursing students in Northern Tanzania. We found that there are existing several facilitators and barriers of effective performance in clinical practice. The facilitating factors are effective supervision, adequate number of tutors, and clinical instructors. Barriers to effective clinical practice included lack of self-confidence, absenteeism, inadequate supervision, lack of resources, and anxiety.

The finding showed that effective supervision which is a facilitator of effective clinical practice is also reported by previous studies [21]. Hand and Schalge [22, 23] reported that effective clinical practice promoted learning and helped students to achieve learning outcomes and competencies through the diversity of learning opportunities. The number of tutors and clinical instructors that match with student numbers could facilitate effective supportive supervision. However it is not the case in resource poor settings where there is a crisis of human resource for health, both in number and in motivation. Existence of barriers to effective clinical practice such as self-confidence, absenteeism, inadequate supervision, lack of resources, and anxiety is reported also in other studies, Awuah-Peasah [24]. This linked to late reporting time and absenteeism from duty leads to poor performance in clinical practice; too many patients may lead to exhaustion and abscondment in clinical areas. Lack of basic equipment and supplies for nursing care procedures makes students ignore clinical practice. The students felt that clinical practice provide better opportunities and favorable setting to apply theory to practice.

Anxiety was reported by majority of the students as one of the factors that affect performance in clinical practice. The finding relate to another study which found that anxiety contributed to poor clinical practice and affected up to 94% female students in the beginning of placements [25]. The experience of anxiety was found to affect more female students than male. Reasons for anxiety were identified as a fear of making mistakes and lack of experience. Effective clinical supervision and giving appropriate patient assignment to students and case presentations of clinical cases could help to reduce anxiety.

In the clinical settings, shortage of staff, lack of learning materials, and overcrowding of patients were important barriers to clinical practice. In some hospitals students could be used to cover shortage of staff instead of meeting the learning outcomes. This finding is similar to a study by Killam and Carter [26] which cited limited resources and shortage of staff which lead to nursing students covering the shortage instead to learning since they were assigned to work similar to qualified nurses. Therefore the hospitals must ensure adequate staffing and resources to support quality clinical training. The perception of facilitators and barriers to clinical practice may relate to how well individual schools prepared students in the skills laboratory and orientation before placement.

## 5. Conclusion

The study revealed several factors that influence performance in clinical practice among preservice diploma nursing students. Three categories of factors were assessed, i.e., students based factors; hospital based factors; and social-economic background of students. Student's factors seemed to play a bigger role in successful clinical learning followed by hospital based factors. Inadequate supervision by clinical instructors, lack of resources, quality of practical assessment in clinical area, and anxiety were some of the factors that hindered effective clinical learning. Student's willingness, competency of the clinical of instructor, and attitudes of staff towards students in clinical area have significance performance in clinical practice. Taking these results into consideration, we suggest offering preclinical orientation to the students and distributing and clarifying clinical learning objectives to students before reporting to clinical placement. Furthermore, supervision of students in clinical practice by nurse tutors is essential. Additionally, this can strengthen clinical instructor-nursing students' relationship and develop and/or build confidence and competence among nursing students.

## 6. Study Limitation

Although this study was set to generate important evidence with regard to factors influencing clinical practice of nursing students, some limitations need to be acknowledged. This study was cross-sectional and descriptive in nature; therefore the findings reflects a snapshot of the reality and could be relevant the study context and similar settings. Since all data were collected through a self-administered questionnaire, the study was prone to response bias [27]. Besides, the study topic could be viewed differently by the participants raising the potential for bias in answering the questionnaires [28].

## Figures and Tables

**Table 1:** Regions, districts, nursing schools, number of students, and nurse tutors.

REGION	DISTRICT	NURSING SCHOOLS	NUMBER OF STUDENTS	NUMBER OF NURSE TUTORS	SAMPLE OF NURSING STUDENTS (10%) FROM EACH SCHOOL	SAMPLE FOR NURSE TUTORS
MWANZA	Nyamagana	Bugando	153	9	15	9
	Sengerema	Sengerema	199	10	20	10

KILIMANJARO	Moshi urban	KCMC	150	12	15	12
	Moshi rural	Kibosho	131	10	13	10
	Rombo	Huruma	120	9	12	9
	Hai	Machame	132	12	13	12

MANYARA	Mbulu	Haydom	125	12	13	12
	Babati	Dareda	220	11	22	11

TOTAL	*8*	*8*	*1230*	*85*	*123*	*85*

**Table 2:** Showing the name of teaching hospitals, type of hospital, ownership, bed capacity, and number of staff.

SN	Name of Hospital	Ownership	Type of Hospital	Bed capacity	Number of staff
1	Bugando	FBO	Zonal Referral Hospital	950	1300

2	Sengerema	FBO	District designated Hospital	300	200

3	KCMC	FBO	Zonal referral hospital	630	1300

4	Kibosho	FBO	District designated Hospital	225	178

5	Huruma	FBO	District designated hospital	300	238

6	Machame	FBO	District designated hospital	200	210

7	Haydom	FBO	Regional referral hospital	400	526

8	Dareda	FBO	District designated hospital	200	203

**Table 3:** Factors related to effective clinical practice by nursing students (n=96).

Response of a participant	Frequency	Percent %
*Clinical placement gives opportunity for learning*		

No	15	15.6
Yes	81	84.4

*Factors that contribute improvement in clinical practice*		

Effective supervision and assessment	31	32.3
Provision of quality care to patients	28	29.2
Self-engagement in clinical practice	24	25.0
Competency gaining in clinical areas	9	9.4
Demand to score high marks in clinical practice	4	4.2

*Barriers of good performance on clinical practice*		

No	22	29.9
Yes	74	70.1

*Reasons for poor clinical practice*		

Lack of communication between staff and Students	47	49.0
Improper clinical supervision	25	26.0
Clinical instructors are not well prepared for the Task	18	18.7
Difficulties in orientation for clinical teaching	6	6.3

*Students behavior can be a barrier to clinical learning *		

No	33	34.4
Yes	66	65.6

*Students behaviors affects clinical practice *		

Lack of self confidence	38	39.6
Absenteeism from clinical area from time to time	31	32.3
Unsafe practice	20	20.8
Overconfidence	7	7.3

**Table 4:** Reasons why clinical placement does not give opportunity for learning (n=15).

Participant reported reason	Frequency	Percent
Shortage nurse tutor or someone to guide in clinical area	9	60.0

Inadequate learning resources	4	26.7

Inadequate supervision	2	13.3

*Total*	*15*	*100.0*

**Table 5:** Causes and measure to reduce anxiety by nursing students (n=96).

Response of a participant	Frequency	Percent
*Intrinsic causes of anxiety in clinical areas*		

Fear of making mistakes	46	47.9
Lack of competency	30	31.2
Lack of experience	14	14.6
Fear of clinical environment	6	6.3

*Situations that cause anxiety in the clinical area*		

Clinical assessment during examination	37	38.5
Strict supervision	25	26.0
Death of patient	22	22.9
Practicing in intensive care unit	12	12.5

*Measures to reduce anxiety*		

Appropriate clinical supervision	43	44.8
Assignment and clinical case presentation	28	29.2
Proper placement in clinical area	18	18.7
Effective classroom teaching	7	7.3

**Table 6:** Factors affecting performance in clinical practice in hospital (n=96).

Response of a participant	Frequency	Percent
*Shortage of staff in the hospital affects clinical learning*		

No	10	10.4
Yes	86	89.6

*Common missing resource that affects clinical practice*		

Lack of competent clinical instructors, tutors and Coordinators	41	42.7
Inadequate physical resources (skills laboratory, library and classrooms)	22	22.9
Lack of teaching facilities in clinical area	21	21.9
Lack of fund for learning materials	9	9.4
Inadequate number of patients	3	3.1

*Factors in clinical area that negatively affect clinical practice*		

Non supportive environment due to many patients	39	40.6
Practicing under stressful situation	25	26.0
Inadequate PPE (personal protective equipment)	25	26.0
Caring of serious patients	7	7.3

**Table 7:** Reasons on how shortage of staff affects clinical learning (n=86).

Participant reported reason	Frequency	Percent
Clinical staff are too busy to an extent that they do not have time to guide students	47	54.7

Students are there to cover shortage instead of learning	17	19.8

Inadequate number of clinical instructors	10	11.6

Improper supervision during the procedures	12	13.9

*Total*	*86*	*100.0*

**Table 8:** Effects of parents' low economic status on clinical practice (n=81).

Participant reported factors	Frequency	Percent
Creates stressful environment which lead to low clinical practice	31	38.3

Failure to buy learning materials and other personal needs	16	19.6

Unwanted pregnancy among female students	12	14.8

Absence from clinical area seeking for fee	11	13.6

Low concentration in the class results in poor Clinical practice.	8	9.9

Failure to report to school on time and later fail in examination	3	3.7

*Total*	*81*	*100*

**Table 9:** Effects of good social environment on clinical practice (n=83).

Participant reported behaviors	Frequency	Percent
Favors sharing of knowledge, experience and Different ideas among students.	20	24.1

Creates good interpersonal relationship and good cooperation among students	17	20.5

Motivates and improves self confidence among students	17	20.5

Reduces unfavorable behavior among students (delinquency, criminal behavior, use of drugs and unwanted pregnancy)	11	13.2

Reduces anxiety	7	8.4

Provide good opportunity for clinical learning, assessment and supervision	6	7.2

Facilitate positive communication between students, teachers and Patients	5	6.0

*Total*	*83*	*100*

**Table 10:** Tutors opinions on ways to improve students' clinical practice (N=85).

Ways to improve students' clinical practices	Frequency	Percent
Recruitment of adequate number of nurse tutors that matches with the ratio of students	44	51.8

Availability of modern skills lab for demonstration and ensure availability of equipment	36	42.4

Train more tutors and to give incentives so that they can be committed	32	37.6

Conduct pre-assessment skills before sending students to clinical area and continuous assessment tests and giving feedback on time	26	30.6

Enrolment of optimum number of students who can be supervised easily	23	27.1

Utilization of bed-side teaching and proper supervision of students	22	25.9

Selection of students with interest in nursing career	21	24.7

Conducting regular follow up of students during clinical practices	20	23.5

Good cooperation between staff and students	18	21.2

Allocate more time in clinical area	15	17.6

Clinical instruction should go hand in hand with the topic covered in the class room	14	16.5

Equip students with theory before sending them to clinical area	13	15.3

Constant supervision of students	7	8.2

## Data Availability

The data used to support the findings of this study are available from the corresponding author upon request.
